# An MRI-Based Radiomic Model for Individualized Prediction of Hepatocellular Carcinoma in Patients With Hepatitis B Virus-Related Cirrhosis

**DOI:** 10.3389/fonc.2022.800787

**Published:** 2022-03-14

**Authors:** Yichen Wei, Jie Gong, Xin He, Bo Liu, Tiejun Liu, Shuohui Yang, Zhipeng Zhou, Lingyan Liang, Songhua Zhan, Ziqiang Xia, Gaoxiong Duan, Bin Lin, Qiuli Han, Shasha Li, Wei Qin, Perry J. Pickhardt, Demao Deng

**Affiliations:** ^1^ Department of Radiology, The People’s Hospital of Guangxi Zhuang Autonomous Region, Nanning, China; ^2^ Life Science Research Center, School of Life Science and Technology, Xidian University, Xi’an, China; ^3^ Department of Radiology, First Affiliated Hospital, Guangxi University of Chinese Medicine, Nanning, China; ^4^ Department of Radiology, Second Affiliated Hospital of Guangzhou University of Chinese Medicine, Guangzhou, China; ^5^ Department of Radiology, Affiliated Hospital, Guangxi Medicine University, Liuzhou People’s Hospital, Liuzhou, China; ^6^ Department of Radiology, Shuguang Hospital Affiliated to Shanghai University of Traditional Chinese Medicine, Shanghai, China; ^7^ Department of Radiology, Affiliated Hospital of Guilin Medical University, Guilin, China; ^8^ School of Medicine and Public Health, University of Wisconsin, Madison, WI, United States

**Keywords:** cirrhosis, hepatocellular carcinoma, radiomics, magnetic resonance imaging, nomogram, prediction

## Abstract

**Objective:**

To develop and validate a radiomic nomogram for individualized prediction of hepatocellular carcinoma (HCC) in HBV cirrhosis patients based on baseline magnetic resonance imaging examinations and clinical data.

**Methods:**

364 patients with HBV cirrhosis from five hospitals were assigned to the training, internal validation, external validation-1 or external validation-2 cohort. All patients underwent baseline magnetic resonance image (MRI) scans and clinical follow-up within three-year time. Clinical risk factors and MRI-based features were extracted and analyzed. The radiomic signatures were built using the radiomics-score (Rad-score) that calculated for each patient as a linear weighted combination of selected MRI-based features. Prognostic performances of the clinical and radiomic nomograms were evaluated with Cox modeling in the training and validation cohorts.

**Results:**

Eighteen features were selected for inclusion in the Rad-score prognostic model. The radiomic signature from multi-sequence MRI yielded a concordance index (C-index) of 0.710, 0.681, 0.632 and 0.658 in the training, internal validation, external validation-1, external validation-2 cohorts, respectively. Sex and Child-Turcotte-Pugh (CTP) class were the most prognostic clinical risk factors in univariate Cox proportional hazards analyses. The radiomic combined nomogram that integrated the radiomic signature with the clinical factors yielded a C-index of 0.746, 0.710, and 0.641 in the training, internal validation, and external validation-1 cohorts, respectively, which was an improvement over either the clinical nomogram or radiomic signature alone.

**Conclusion:**

We developed an MRI-based radiomic combined nomogram with good discrimination ability for the individualized prediction of HCC in HBV cirrhosis patients within three-year time.

## 1 Introduction

Hepatocellular carcinoma (HCC), a primary liver cancer, is the sixth-most common cancer and the second-leading cause of cancer-related deaths worldwide (1). Hepatitis B virus (HBV) cirrhosis induces a scarring of the liver and is the primary cause of HCC in the Asian-Pacific region (2). The five-year survival rate is higher among patients with early-stage HCC compared with those presenting with advanced HCC (3). Thus, early diagnosis and intervention for HCC are critical for improving the prognosis of cirrhotic patients.

In recent years, there has been a number of studies intent on predicting HCC in patients with chronic liver diseases, which have used various risk-scoring systems that combine clinical symptoms and laboratory variables (4, 5), liver stiffness measurements by ultrasound, and MRI-based elastography measurements (6). Despite this existing research, there are limitations as it pertains to the predictive value and assessment of HCC in cirrhotic patients. No single risk scoring model is universally accepted because of unsatisfactory validation across geographic regions, and MRI-based elastography only allows for assessment of fibrosis and cirrhosis *via* estimation of liver stiffness (7, 8). Liver biopsy is considered to be the reference standard for fibrosis, but is not an ideal tool for screening because of its invasiveness, cost, complications, and sampling error. One common limitation with these predictive hepatocarcinogenic measurements is that all HCC predictions have been based on the risk factors of chronic hepatitis, not cirrhosis specifically. The risk of developing HCC in patients with cirrhosis is not the same as patients without cirrhosis (1). As a widely validated non-invasive tool, MRI is the most powerful imaging method to detect early liver cirrhosis and HCC in various chronic liver diseases (9). Previous studies have shown that contrast-enhanced T1-weighted imaging alone has the ability to diagnose HCC in cirrhosis, without the need for biopsy (10). MRI could also be used to diagnose cirrhosis and grade liver fibrosis on the basis of hepatic textural alterations after the administration of MR contrast agents (11, 12). However, few if any studies have provided accurate hepatocarcinogenic prediction in cirrhotic patients using MR images.

Radiomics exploits sophisticated image analysis tools handling medical imaging data to improve diagnostic, predictive, and prognostic accuracy in cancer research, providing a powerful tool in modern medicine (13–18). Radiomics detects high-dimensional, heterogeneous imaging features objectively and quantitatively, and may have the potential to help predict clinical outcomes, has proven to be able to predict clinical outcomes based on anatomical and functional MRI (19). Recently, elastography displayed excellent performance for assessing cirrhosis by using the developed deep learning radiomics, with similar diagnostic efficacy with the liver biopsy (13). To the best of our knowledge, our study represents the first study to use MRI -based radiomics to predict hepatocarcinogenic durations in patients with cirrhosis. The aim of this study was to develop and validate a radiomic model for the individualized prediction of HCC occurrence in HBV cirrhosis patients based purely on baseline MRI and clinical data.

## 2 Materials and Methods

### 2.1 Patients

The MRI and clinical data of 11,519 patients were retrospectively analyzed from March 2011 and August 2018 at five different hospitals, of which 364 patients with HBV cirrhosis were considered eligible and selected for inclusion to the training, internal validation, external validation-1 or external validation-2 cohort. The inclusion criteria were: (1) Cirrhosis diagnosed by clinical diagnosis and typical features on conventional MRI; (2) Cirrhosis caused by hepatitis B virus infection; (3) No typical or suspicious HCC on initial baseline MRI examination; (4) Complete related clinical information (e.g., age, gender, family history of HCC, serum HBV-DNA, CTP class, serum alpha fetoprotein (AFP), alcohol consumption, and smoking) - for training, internal validation, and external validation-1 cohorts. The exclusion criteria were: (1) Cirrhosis caused by other factors beyond hepatitis B (e.g., drug-induced cirrhosis, alcoholic cirrhosis, hepatitis C cirrhosis, chronic passive hepatic congestion); (2) HCC diagnosed on the initial baseline MRI examination, or with prior history of HCC or liver surgery; (3) Insufficient MRI quality to obtain measurements (e.g., owing to motion artifacts, massive ascites); (4) Lack of related clinical information for training, internal validation, and external validation-1 cohorts.

All participants were informed about the experimental procedure and signed a written informed consent form. The study was approved by the local Medicine Ethics Committee.

### 2.2 MRI Data Acquisitions

Data from the initial baseline MRI were selected from the patients with HBV cirrhosis, to be used for radiomic feature extraction and radiomic signature building. Specific sequences utilized included the T1-weighted pre-contrast, hepatic arterial phase (HAP), portal venous phase (PVP) and equilibrium phase (EP) of contrast enhancement and T2-weighted images for each patient. A transverse breath-hold three-dimensional T1-weighted fat-suppressed spoiled gradient recalled volumetric interpolated breath-hold examination (Siemens) or enhanced-T1 high resolution isotropic volume examination (Philips) sequence before and after injection of 0.1 mmol per kilogram of body weight of contrast material (gadolinium diethylenetriaminepentaacetic acid) followed by a 20 ml saline flush (2.5 ml/s) with a high-pressure injector. The triple-phase (HAP, PVP, and EP) of contrast enhancement axial T1-weighed fat-suppressed images were obtained at 20, 70, and 180 seconds. The MR examinations were performed from the five hospitals. See **Supplementary Table 1** for the scanner and protocol details.

### 2.3 Clinical Factors

Clinical factors and baseline MRI data acquisition were collected within two weeks. Clinical factors included age, sex, family history of HCC, serum HBV-DNA, Child-Turcotte-Pugh (CTP) classification, serum alpha fetoprotein (AFP), alcohol consumption, and smoking. See **Supplementary Material 1.1** for more details. The clinical data of patients in the external validation-2 cohort was more limited, so the clinical nomogram assessment for this group was precluded.

### 2.4 HCC Surveillance and Diagnosis

**Figure 1:**
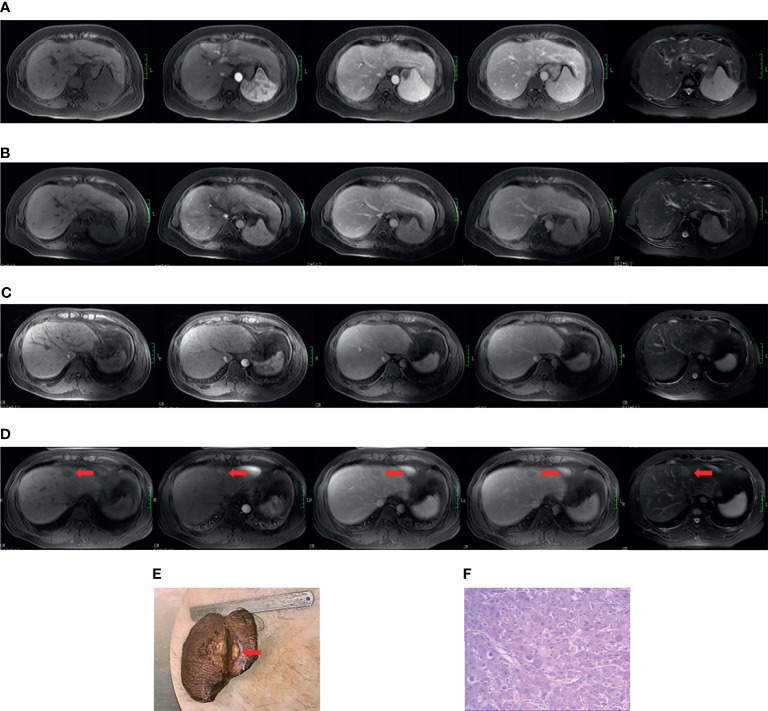
Case examples of the non-HCC and positive HCC subgroups. Case 1 **(A, B)**. MR images from a 49-year-old cirrhotic patient in the non-hepatocarcinogenic group. Axial fat-suppressed T1- and T2-weighted images show a cirrhotic liver morphology appears cirrhotic. Innumerable regenerative nodules are present throughout the liver, without suspicious nodules. **(A)** No HCC was found on the baseline MRI examination. **(B)** Similarly, no HCC lesion was seen after final follow-up at 70 months. Case 2 **(C–F)**. MR images from a 34-year-old man in hepatocarcinogenic group. Axial fat-suppressed T1- and T2-weighted images show a cirrhotic liver with nodular contour. **(C)** Innumerable regenerative nodules were seen throughout the liver on baseline MRI examination. **(D)** At 16-month follow-up MRI, a new nodule about 3.1cm×2.0cm×2.0cm in size was clearly seen (red arrows) in segment four. The nodule appears hyperintense on T2-weighted image and hypointense on the pre-contrast T1-weighted images, with diagnostic enhancement of the arterial phase and subsequent washout on the portal venous and equilibrium phases. In addition, the tumor is surrounded by a fibrous capsule on the portal venous and equilibrium phases. **(E)** The liver explant specimen shows the hepatocellular carcinoma (red arrows) adjacent to Glisson’s capsule. **(F)** At histopathology (original magnification×40, hematoxylin-eosin staining), a moderately differentiated hepatocellular carcinoma is confirmed.

The diagnosis of cirrhosis was according to typical features demonstrated by conventional MRI (20). The diagnostic criteria for HBV cirrhosis was according to the guidelines of prevention and treatment for chronic hepatitis B (2019 version) (21). The diagnosis of HCC was according to the 2018 American Association for the Study of Liver Diseases (AASLD) guidelines (22, 23). See **Supplementary Material 1.2** for the complete cirrhosis diagnostic standards. Two cases of cirrhosis from the non-HCC and positive HCC subgroups are presented in **Figure 1**.

### 2.5 Follow-Up and Clinical Endpoint

The MRI follow-up interval of all patients with HBV cirrhosis was 3-12 months. The endpoint of the hepatocarcinogenic group was the occurrence of HCC diagnosed by MRI examination and/or histopathological confirmation, while the required follow-up time of non-hepatocarcinogenic group was 36 months or greater. Hepatocarcinogenic time was defined as the time from the first/baseline MRI examination with cirrhosis until HCC diagnosis by MRI examination and/or histopathological confirmation. The flow diagram of HCC surveillance and diagnosis of patients with HBV cirrhosis are shown in **Supplementary Figure 1**.

### 2.6 Data Processing

#### 2.6.1 Image Pre-Processing and Liver Segmentation

**Figure 2:**
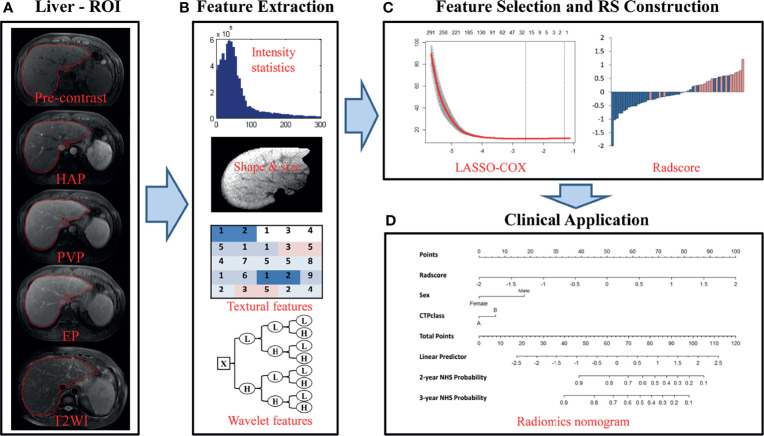
Flowchart of the study. **(A)** Liver segmentation is performed on axial T2-weighted and contrast-enhanced T1-weighted imaging. Pre-contrast, pre-contrast phase; HAP, hepatic arterial phase; PVP, portal venous phase; EP, equilibrium phase; T2WI, T2 weighted imaging. **(B)** Four types of features are extracted from within the defined liver areas on the MR images, including quantify intensity, shape, texture and wavelet texture of the entire liver. **(C)** The LASSO method was applied to select suitable features and the selected features were used to calculate the Rad-score for the radiomic nomogram. **(D)** The radiomic signature incorporated the prognostic clinical risk factors in a final radiomic nomogram for individual evaluation.

Prior to liver segmentation, images were pre-processed. First, images were co-registered using FLIRT (FMRIB’s Linear Image Registration Tool) from the FMRIB Software Library (FSL, www.fmrib.ox.ac.uk/fsl) and intensities were normalized using a post-processing method (24). Specifically, T1-weighted images of patients were registered to corresponding T2-weighted images for motion correction. MRI intensities in each MRI protocol were normalized so that scans of all patients in the same protocol had a similar intensity distribution, which could generate well-defined inputs for liver segmentation and data processing. The images of the other four hospitals were resampled according to the standards of First Affiliated Hospital of Guangxi University of Chinese Medicine to reduce the impact of the difference between the scanner and protocol details of the five hospitals on the results. Liver segmentation was performed by three radiologists who were blinded to the clinical and/or pathological data (**Figure 2A**). The whole livers were delineated as the regions of interest (ROIs) *via* the ITK-SNAP software (www.itksnap.org) by one radiologist with five years of clinical-diagnosing experience. The whole liver was manually drawn on each slice using the T1- weighted contrast-enhanced three-phase images and T2-weighted data of each patient. The other two radiologists with 10 years of experience corrected the ROIs by consensus.

Thirty patients from First Affiliated Hospital of Guangxi University of Chinese Medicine were randomly selected to analyze the inter- and intra-observer reproducibility of liver delineation and radiomic feature extraction. To ensure reproducibility, each radiologist followed the same procedure twice with an interval of at least 1 week to repeat the tumor masking and generation of radiomic features. Intra-class correlation coefficients (ICCs) were used to evaluate the intra- and inter-observer agreement in terms of feature extraction. We interpreted an ICC of 0.81-1.00 as almost perfect agreement, 0.61-0.80 as substantial agreement, 0.41-0.60 as moderate agreement, 0.21-0.40 as fair agreement, and 0-0.20 as poor or no agreement (25). An ICC above 0.6 was considered a mark of satisfactory inter- and intra-observer reproducibility.

#### 2.6.2 Radiomic Feature Extraction/Selection and Radiomic Signature Building

In total, 4,282 imaging features were extracted and analyzed from the manually segmented liver MR images from each sequence for every patient with MATLAB (Mathworks, Natick, MA, USA) using a developed in-house toolbox and included four groups: (i) 13 intensity statistics features calculated from the intensity histogram; (ii) 7 shape- and size-based features; (iii) 462 textural features based on gray level co-occurrence texture matrices (GLCM) and gray level run-length texture matrices (GLRLM); and (iv) 3,800 wavelet features(8 wavelet-decomposed images, 13*8+462*8 = 3800) (**Figure 2B**). All features have been described in previous studies and were in accordance with feature definitions as described by the IBSI (14, 26–28). Five sequences (axial fat-suppressed T2-weighed images, pre-contrast, arterial, portal venous and equilibrium phase images) resulted in a total of 21,410 radiomic features per patient. To reduce any type of bias or over-fitting caused by too many features, we used the least absolute shrinkage and selection operator (LASSO) method to select features that were most significant and then built a Cox model in the training cohort based on each unimodal sequence and multimodal sequence(**Figure 2C**) (29). Most of the coefficients of the covariates were reduced to zero and the remaining non-zero coefficients were selected by LASSO. The Rad-score was calculated for each patient as a linear combination of selected features that were weighted by their respective coefficients. Radiomic signatures were built using the Rad-score (19).

#### 2.6.3 Prognostic Validation of Radiomic Signature

Univariate Cox proportional hazards models were applied to calculate the C-index of the radiomic signatures from five unimodal MRI sequences and for the combined multi-sequence MRI for prediction of non-HCC survival (over 36 months) in the training and validation cohorts. The potential association of the radiomic signature from the combined multi-sequence MRI for prediction of non-HCC survival was first assessed in the training cohort, and then subsequently validated in each of the validation cohorts by using Kaplan-Meier survival analysis. The patients were divided into high-risk and low-risk groups based on the optimal cutoff value of the Rad-score, which was based on the score from the Cox regression model. Then, the same threshold values were applied to the validation cohorts. We performed stratified analyses to determine non-HCC survival in various subgroups for clinical risk factors to compare high-risk and low-risk patients.

#### 2.6.4 Assessment of Incremental Value of Radiomic Signature in Individual HCC Prediction

To demonstrate the incremental value of the radiomic signature to the clinical risk factors for individualized assessment of non-HCC survival, both a radiomic model and a clinical model were developed by multivariable Cox proportional hazard regression analyses in the training cohort. The radiomic combined nomogram integrated the radiomic signature and the prognostic clinical risk factors into the multivariable Cox proportional hazards model (**Figure 2D**). The clinical model integrated only the prognostic clinical risk factors into the model. The performance of the model was measured quantitatively using the C-index. The C-index is commonly used to evaluate the discriminative ability of prognostic models in survival analysis. The value of the C-index can range from 0.5, which indicates no discriminative ability, to 1.0, which indicates a perfect ability to distinguish between patients who experience disease progression or death from those who do not. Bootstrap analyses with 1,000 resamples were used to obtain C-index statistics that were corrected for potential overfitting. The prognostic performances of the clinical model and radiomic model were evaluated in the training cohort and then tested in the validation cohort. The performance of the radiomic model was compared with that of the clinical model by likelihood ratio test (30). To provide the clinician with a quantitative tool for individualized assessment of HCC prediction, we built the radiomic nomogram on the basis of the multivariable Cox proportional hazards model in the training cohort. The nomogram calibration curves were assessed by plotting the observed survival fraction against the nomogram-assessed probabilities.

#### 2.6.5 Statistical Analysis

Statistical analysis of the radiomic signature was performed using the following R packages in R statistical software version 3.3.3 (http://www.R-project.org). The “glmnet” package was used to perform the LASSO Cox regression model analysis. The “survival” package was used for Kaplan–Meier survival analyses. The “rms” package was used for Cox proportional hazards regression, nomograms, and calibration curves. The “cutp” function of the “survMisc” package was used to calculate the optimal cutoff value of the Rad-score. The Hmisc package was used for comparisons between C-indices. The Resource Selection package was used to apply Hosmer–Lemeshow tests. All statistical tests were two-sided, and p-values <0.05 were considered significant.

## 3 Results

### 3.1 Clinical Characteristics

In total, 364 patients with HBV cirrhosis were deemed eligible and selected for the four cohorts in this study. 163 and 56 patients were randomly assigned to the training cohort and internal validation cohort, respectively. 91 and 54 patients were respectively assigned to external validation-1 cohort and external validation-2 cohort. Demographic and clinical characteristics for these cohorts are listed in **Table 1**. A total of 131 (36.0%) patients had an HCC occurrence within the follow-up period, including 61 patients from the training cohort, and 21, 26, and 24 patients from the internal validation cohort, external validation-1 cohort, and external validation-2 cohort, respectively. The remaining 233 patients had non-hepatocarcinogenesis within the follow-up time (non-HCC survival). There were no significant differences in the clinical characteristics between the four cohorts. For HCC prediction, we defined sex and CTP class, which both reached p-values less than 0.05 in the univariate Cox proportional hazards analyses, as the main prognostic clinical risk factors and used them for further analyses. The results of univariate Cox proportional hazards analyses for all clinical factors are listed in **Supplementary Table 2**.

**Table 1:** Characteristics of patients in the training and validation cohorts.

Characteristic	Training cohort (N=163)	Internal Validation cohort(N=56)	1^st^ External Validation cohort(N=91)	2^nd^ External Validation cohort(N=54)	p-value
**Age (years)**	51 (44–61)	52 (46-63)	51 (44-59)	57 (47-63)	0.254
Median (IQR)					
**Follow-up (months)**	36 (18-44)	38 (14-52)	39 (33-45)	36 (28-57)	0.305
Median (IQR)					
**Gender**					
Male	129 (79.1%)	45 (80.4%)	61 (67.0%)	39 (72.2%)	0.128
Female	34 (20.9%)	11 (19.6%)	30 (33.0%)	15 (27.8%)	
**FH of HCC^a^**				**-**	0.594
Yes	16 (9.8%)	3 (5.4%)	8 (8.8%)		
No	147 (90.2%)	53 (94.6%)	83 (91.2%)		
**HBV-DNA level**				**-**	0.459
<10^4^	102 (62.6%)	40 (71.4%)	57 (62.6%)		
≥10^4^	61 (37.4%)	16 (28.6%)	34 (37.4%)		
**AFP**	6.0 (3.0-12.0)	4.0 (3.0-13.25)	7.0 (3.0-12.0)	-	0.690
Median (IQR)					
**CTP class^b^**				**-**	0.522
A	142(87.1%)	44(78.6%)	76 (83.5%)		
B or C	21(12.9%)	12(21.43%)	15 (16.5%)		
**Drinking**				**-**	0.156
<40g/d	138 (84.7%)	45 (80.4%)	83 (91.2%)		
40-80g/d	25 (15.3%)	11 (19.6%)	8 (8.8%)		
**Smoking**				**-**	0.148
No	134 (82.2%)	48 (85.7%)	83 (91.2%)		
Yes	29 (17.8%)	8 (14.3%)	8 (8.8%)		

Chi-Square or Fisher Exact tests, as appropriate, were used to compare the differences in categorical variables (Gender, FH of HCC, Serum HBV-DNA level, CTP class, Drinking, Smoking), while Kruskal-Wallis test was used to compare the differences in age, Serum AFP, and Follow-up. The value for Serum HBV-DNA level (<10^4^&≥10^4^), CTP class (A vs. B/C), Drinking alcohol (<40g/d and 40-80g/d), and smoking (non-smoking vs. smoking) were according to previous studies. FH of HCC^a^, family history of HCC; CTP class^b^, Child-Turcotte-Pugh classification. Clinical data were lacking for the external validation-2 cohort.

### 3.2 Radiomic Feature Extraction/Selection and Radiomic Signature Building

A total of 21,410 imaging features were extracted from the manually segmented liver MR images from the five sequences from each patient. Satisfactory inter- and intra-observer reproducibility of tumor masking and radiomic feature extraction was achieved with ICC>0.6 both among the masks from the all radiologists at baseline and between the masks from the same radiologist at baseline and at least 1 month later.

**Figure 3:**
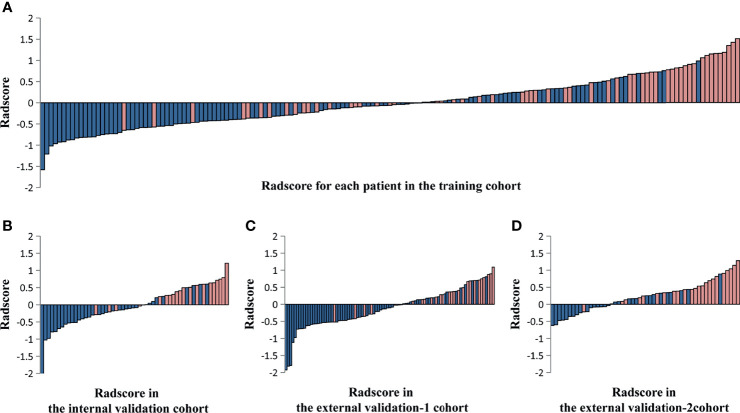
The Rad-score calculated from multi-sequence MRI including four-phase (precontrast, arterial, portal venous, and equilibrium phases) and T2-weighted series for each patient in the training cohort and the three validation cohorts. Rad-score for each patient in the training cohort **(A)**, the internal validation cohort **(B)**, the external validation-1 cohort **(C)**, and the external validation-2 cohort **(D)**. Blue bars show scores for patients who survived at least 36 months without hepatocarcinogenesis (non-HCC survival), while pink bars show scores for those who developed hepatocarcinogenesis (positive HCC diagnosis).

Of these, five radiomic signatures from unimodal (single sequence) MRI including four-phase (precontrast, arterial, portal venous, and equilibrium phases) and T2-weighted series were further constructed. To build the radiomic signature from multi-sequence MRI (combined sequences), 18 features were selected from all features for inclusion in the Rad-score prognostic model (**Supplementary Table 3**). The Rad-score calculated from multi-sequence MRI for each patient in the training cohort and the three validation cohorts is shown in **Figure 3**.

### 3.3 Validation of Radiomic Signature

**Figure 4:**
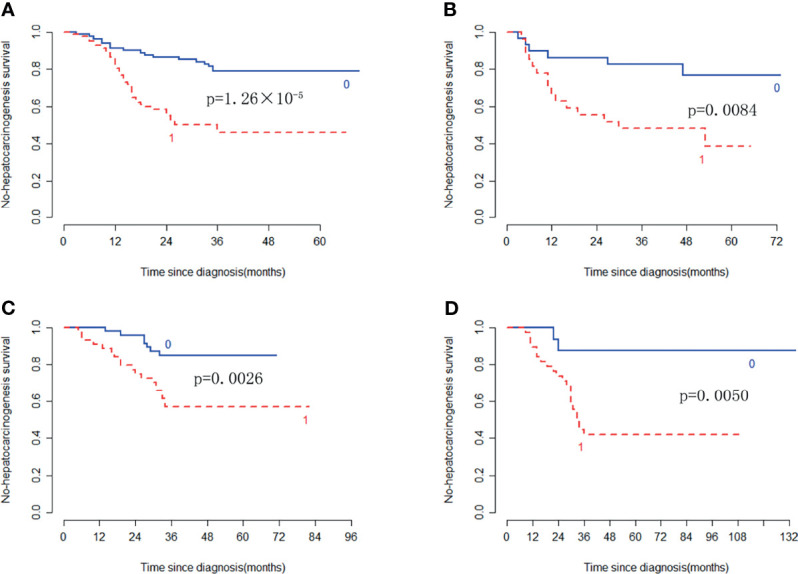
Kaplan-Meier survival analysis of the high-risk and low-risk patients for developing HCC in the training cohort and the three validation cohorts. Significant discrimination between the high-risk (red-dashed line [1]) and low-risk (blue line [0]) patients for developing HCC by using the Rad-score in the training cohort **(A)**, the internal validation cohort **(B)**, the external validation-1 cohort **(C)**, and the external validation-2 cohort **(D)**.

**Figure 5:**
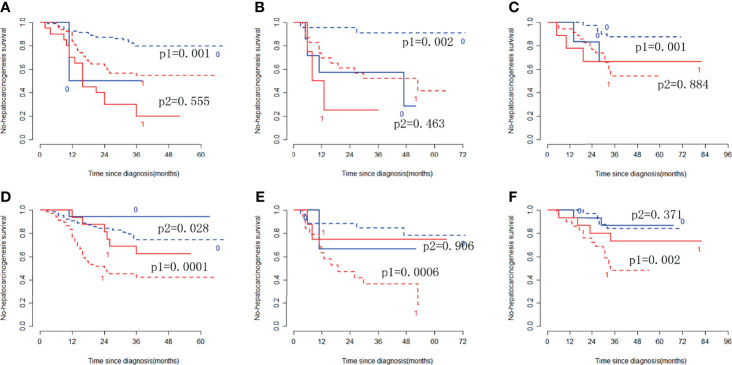
Stratified analyses were performed to estimate non-HCC survival in various subgroups, comparing low-risk patients [blue lines (0)] and high-risk patients [red lines (1)]. Stratified analyses for CTP class [type A, dotted lines (p1); type B/C, solid lines (p2)] for the training cohort **(A)**, internal validation cohort **(B)**, and external validation cohort **(C)**. Stratified analyses for sex [Male, dotted lines (p1); Female, solid lines (p2)] in the training cohort **(D)**, internal validation cohort **(E)**, and external validation cohort **(F)**.

In the training cohort, the radiomic signature from multi-sequence MRI yielded the highest C-index, which was 0.710 (95% confidence interval (CI): 0.635-0.784). In the internal validation cohort, external validation-1 cohort, and external validation-2 cohort, the radiomic signature from multi-sequence MRI yielded a C-index of 0.681 (95% CI: 0.549-0.813), 0.632 (95% CI: 0.518-0.746), and 0.658 (95% CI: 0.535-0.781), respectively. The C-index of radiomic signatures from unimodal MRI is shown in **Table 2**. The performance of each T1-weighted sequence was better than that of the T2-weighted sequence. The performance of multi-sequence MRI was better than that of unimodal MRI. A log-rank test was used to select the Rad-score with statistical significance. The Rad-score value of -0.0586 was used to divide patients into high-risk and low-risk HCC groups. Kaplan-Meier survival analysis showed that the low-risk group with the lower Rad-score had a significantly better non-HCC survival than that of the high-risk group with a high Rad-score (p = 1.26×10^-5^, **Figure 4A**) in the training cohort; and the result was subsequently validated in the internal validation cohort (p = 0.0084 **Figure 4B**), the external validation-1 cohort (p = 0.0026 **Figure 4C**), and the external validation-2 cohort (p = 0.0050 **Figure 4D**). Significant discrimination for non-HCC survival in high-risk and low-risk patients was observed in the prognostic clinical risk factors when subgroup analyses were performed **Figure 5**.

**Table 2:** C-index of radiomic signatures from unimodal (single-sequence) MRI and multi-modality (combined sequences) MRI for prediction of non-HCC survival at 3 years.

Modality	Training	Internal validation	External validation-1	External validation-2
**pre-contrast**	0.680 (0.548-0.812)	0.668 (0.536-0.800)	0.492 (0.378-0.606)	0.416 (0.293-0.539)
**HAP**	0.699 (0.625-0.775)	0.672 (0.540-0.804)	0.526 (0.412-0.640)	0.475 (0.352-0.598)
**PVP**	0.691 (0.616-0.766)	0.654 (0.523-0.796)	0.586 (0.472-0.699)	0.440 (0.317-0.562)
**EP**	0.691 (0.616-0.766)	0.646 (0.514-0.777)	0.505 (0.391-0.619)	0.421 (0.298-0.544)
**T2WI**	0.668 (0.593-0.743)	0.604 (0.472-0.736)	0.478 (0.365-0.592)	0.591 (0.468-0.714)
**ALL**	**0.710 (0.635-0.784)**	**0.681 (0.549-0.813)**	**0.632 (0.518-0.746)**	**0.658 (0.535-0.781)**

The bold values indicate that the performance of the model based on multi-modality (combined sequences) MRI is the best. pre-contrast, pre-contrast phase; HAP, hepatic arterial phase; PVP, portal venous phase; EP, equilibrium phase; T2WI, T2 weighted imaging.

### 3.4 The Incremental Value of the Radiomic Signature When Added to the Clinical Data in the Training Cohort and Validation of the Nomograms

**Figure 6:**
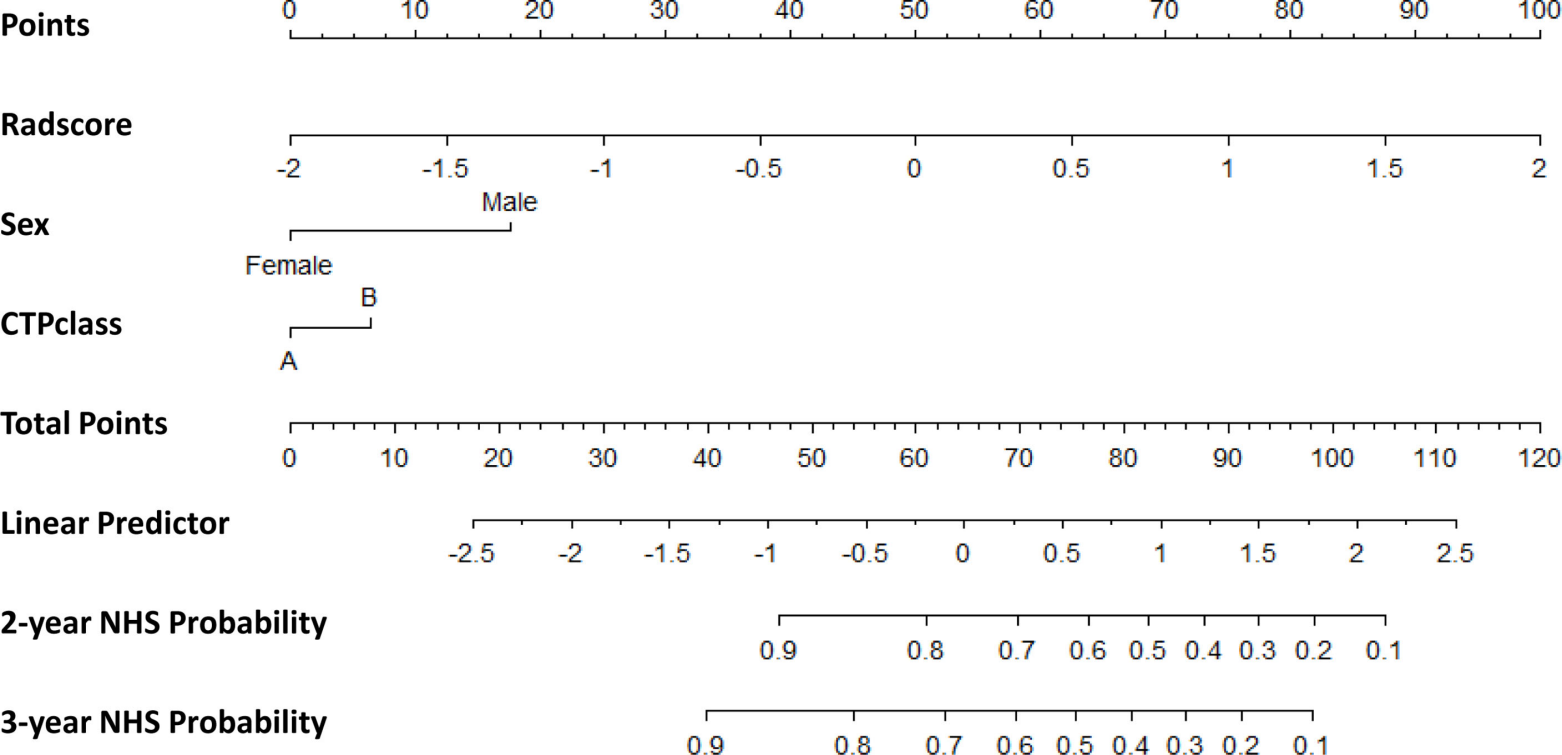
The radiomic nomogram developed, tested, and validated in this study—including Rad-score and key clinical variables (sex and CTP class) —for two- and three-year non-HCC survival (NHS) in patients with HBV cirrhosis. The nomogram allows the user to obtain the probability of two- and three-year non-HCC survival corresponding to a patient’s combination of covariates. As an example, one can locate the patient’s sex and draw a line straight upward to the “Points” axis to determine the score associated with that level of sex. One can then repeat the process for each variable, and sum the scores achieved for each covariate, and locate this sum on the “Total Points” axis. Then, one can draw a line straight down to determine the likelihood of the two- and three-year non-HCC survival.

**Figure 7:**
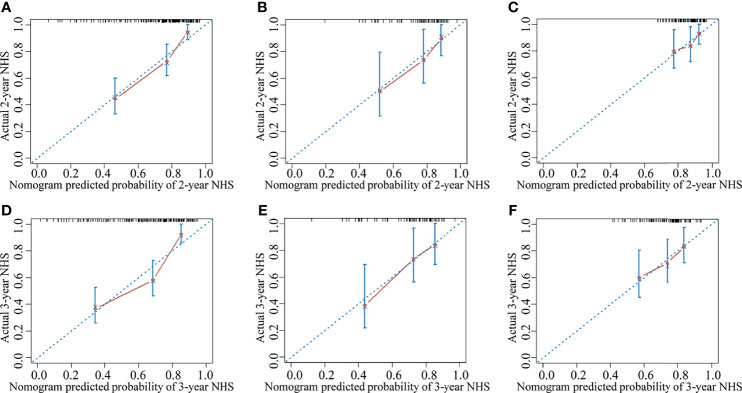
The calibration curve for the probability of non-HCC survival showed good agreement between the nomogram-evaluated and actual survival at two and three years in the training cohort and the validation cohorts. The calibration curve of the radiomic nomogram for predicting non-HCC survival at **(A)** two years and **(D)** three years in the training cohort, at **(B)** two years and **(E)** three years in the internal validation cohort, and at **(C)** two years and **(F)** three years in the external validation-1 cohort. Actual non-HCC survival (NHS) is plotted on the y-axis; nomogram-predicted probability of non-HCC survival is plotted on the x-axis. The solid line represents the performance of the radiomic model. Dashed line indicates a perfect prediction by an ideal model. A closer fit of the solid line to the dashed line represents a better prediction.

The clinical nomogram in the training cohort, including sex and CTP class, yielded a C-index of 0.639 (95% CI: 0.577-0.701). We created a radiomic nomogram that integrated the radiomic signature from multi-sequence MRI with the prognostic clinical risk factors (**Figure 6**) and found that the final model provided a C-index of 0.746 (95% CI: 0.672-0.822) and showed strong calibration (**Figures 7A, D**). Hence, the radiomic nomogram appeared to be more accurate than the clinical nomogram for evaluating HCC risk (p = 6.175×10^-7^).

The results were then tested in the internal validation cohort and the external validation-1 cohort (clinical data were lacking for the external validation-2 cohort, precluding assessment). The clinical nomogram yielded a C-index of 0.640 (95% CI: 0.532–0.748) and 0.562 (95% CI: 0.461–0.662). The radiomic nomogram that integrated the radiomic signature with the clinical factors showed an improvement over the clinical nomogram alone (C-index: 0.710; 95% CI: 0.578-0.842) and 0.641 (95% CI: 0.527-0.755). The calibration curve for the probability of non-HCC survival showed good agreement between the nomogram-evaluated and actual survival (**Figure 7**).The C-index of the clinical model and the radiomic combined nomogram for prediction of non-HCC survival in the primary cohort and validation cohorts are listed in **Table 3**.

**Table 3:** C-index of the clinical model and combined nomogram for prediction of non-HCC survival.

Model	Training	Internal validation	External validation-1
**Clinical model**	0.639 (0.577-0.701)	0.6400 (0.532-0.748)	0.562 (0.461-0.662)
**Combined nomogram**	0.746 (0.672-0.822)	0.710 (0.578-0.842)	0.641 (0.527-0.755)

Combined nomogram integrated the radiomic signature with the clinical factors.

## 4 Discussion

In this study, we developed and validated a radiomic nomogram that integrated a MRI-based radiomic signature with prognostic clinical risk factors to predict HCC in HBV-related cirrhosis. The MRI-based radiomic signature was extracted large volumes of quantitative features from conventional MR images. The clinical risk factors were sex and CTP class of cirrhosis in this study.

In the study, the performance of each T1-weighted sequence was better than that T2-weighted sequence, and the radiomic signature from multi-sequence MRI yielded the highest C-index, better than that of single-sequence. Multi-sequence MRI provides more information about signals and blood supply of cirrhosis than single-sequence in clinical practice and the results reflect this trend. It is well known that multifocal HCC is frequently found, including multicentric carcinogenesis with multiple independent neoplasms or intrahepatic metastases from a single cancer (31). MR elastography has shown excellent performance for assessing fibrosis and cirrhosis by also incorporating deep learning radiomics in recent study (13). LASSO is designed to avoid overfitting and is suitable to analyze a large mass of radiomic features with a relatively small sample, which makes this model easier to interpret and allows for the identification of features strongly associated with hepatocarcinogenesis (32). Radiomics makes it possible to use MRI to predict HCC in cirrhotic patients by using LASSO in the study. The Rad-score value of -0.0586 was used to divide patients into high-risk and low-risk HCC groups when subgroup analyses were performed in the prognostic clinical risk factors. Furthermore, the radiomic signature successfully classified patients according to their risk for development of HCC.

The risk of hepatocarcinogenesis in patients with chronic HBV infection varies according to age, sex, degree of liver damage, level of viral replication, and a family history of HCC. In this study, being male was an independent risk factor for hepatocarcinogenesis in HBV cirrhosis. Previous studies have shown that, men on average consume more alcohol, smoke more cigarettes, and have increased iron stores (33). Furthermore, the estrogen signaling pathway, reduced adiponectin levels, and overexpressed sex-determining region on Y chromosome are also associated with the increased incidence of male hepatocarcinogenesis (34–36). The CTP class of cirrhosis was another independent risk factor for HCC development in our study. The reason may be that the relative enhancement of triple-phase MR images in patients with CPT A cirrhosis was significantly higher than patients with CPT B or C cirrhosis. High levels of HBV DNA are known to be another major risk factor for HCC. As such, antiviral therapy could reduce the risk of developing cirrhosis and HCC (37). However, HBV DNA load was not an independent clinical rick factor for predicting HCC in our study. It concerned to the regularity and exact dosage of patients received antiviral treatment cannot be accurately tracked in our study. Previous studies have shown that the potential for serum AFP levels in predicting HCC is mitigated by individual variation of AFP levels, related to genetic variations and heredity factors (38, 39). In this study, AFP was not an independent clinical rick factor for predicting hepatocarcinogenesis in patients with HBV cirrhosis.

In this study, the combined nomogram model was composed of MRI signatures and some basic clinical risk factors, which showed improved discrimination performance over the clinical risk factors alone in both the training and validation cohorts. Of the currently available prediction tools, the nomogram approach can provide high accuracy and excellent discrimination capabilities for predicting relevant outcomes (40). One previously developed nomogram demonstrated discrimination characteristics for predicting hepatocellular carcinoma risk, which is highly correlated with the corresponding actual risks (41). This combined nomogram would allow clinicians to consider the calculated probability of predicting HCC, along with their own clinical experience in order to make an informed decision with the patient for a rational surveillance plan. Hence, MR images and the clinical risk factors play an important role in the diagnosis and monitoring of liver cirrhosis and HCC. Some literature has also reported that radiomic signature integration of clinical data into radiomic algorithms can improve the accuracy of tumor prediction and personalized medicine (42).

We acknowledge some limitations to our study. First, recognized limits inherent to any retrospective cohort study would apply. Selection bias is inevitable, because the atypical or early cirrhosis patients lack of typical MRI features or not biopsy-proven were not included in our study. Second, since it is difficult to confirm the precise location of carcinogenesis, the entire liver was manually selected as the region of interest in MR images circumvent large vessels and bile ducts in this study. The ROIs manually drawn on each slice of MR images were very time consuming, semi-automatic or automatic drawn will be used in future studies. Third, the C-index of the radiomic combined model was not higher than 0.8, but this MRI-based nomogram provided a novel, non-invasive method for the individualized prediction of HCC in cirrhotic patients. We will increase the amount of data, add the variety of data (e.g. using imaging genomics), and unify MR acquisition parameters to improve reliability of the model in the future prospective study. Additionally, there were some unavoidable aspects that would slightly affect the calculated hepatocarcinogenic time. For example, the MR follow-up interval was variable at 3-12 months, and not unified. Suspicious liver nodules required the development of diagnostic MR features of HCC, which may be delayed until subsequent follow-up. Finally, the entire patient cohort, although derived from multiple centers, was comprised solely of HBV cirrhosis patients. So, the application of radiomics at earlier stages of liver fibrosis and other causes of cirrhosis should be validated in future studies.

## 5 Conclusion

This study developed, tested, and validated a straightforward combined nomogram based on HBV-cirrhosis MRI data and key clinical risk factors. This MRI-based nomogram provided a novel, non-invasive method for the individualized prediction of HCC development in cirrhotic patients.

## Data Availability Statement

The original contributions presented in the study are included in the article/**Supplementary Material**. Further inquiries can be directed to the corresponding authors.

## Ethics Statement

The studies involving human participants were reviewed and approved by the Medicine Ethics Committee of First Affiliated Hospital of Guangxi University of Chinese Medicine, the Medicine Ethics Committee of Second Affiliated Hospital of Guangzhou University of Chinese Medicine, the Medicine Ethics Committee of Liuzhou People’s Hospital, the Medicine Ethics Committee of Shuguang Hospital Affiliated to Shanghai University of Traditional Chinese Medicine and the Medicine Ethics Committee of Affiliated Hospital of Guilin Medical University. The patients/participants provided their written informed consent to participate in this study.

## Author Contributions

YW, JG, and XH performed the study and drafted the manuscript. BLiu, TL, SY, ZZ, and PP was involved in manuscript editing. LL, SZ, ZX, GD, BLin, QH, and SL performed data analysis. WQ and DD designed the study. All authors read and approved the final manuscript.

## Funding

This work was supported by the National Natural Science Foundation of China (Grant Nos. 81760886, 82060315, 82102032) and the Guangxi Natural Science Foundation (Grant Nos. 2016GXNSFAA380086).

## Conflict of Interest

The authors declare that the research was conducted in the absence of any commercial or financial relationships that could be construed as a potential conflict of interest.

## Publisher’s Note

All claims expressed in this article are solely those of the authors and do not necessarily represent those of their affiliated organizations, or those of the publisher, the editors and the reviewers. Any product that may be evaluated in this article, or claim that may be made by its manufacturer, is not guaranteed or endorsed by the publisher.
