# Supplementary material for: An MRI-Based Radiomic Model for Individualized Prediction of Hepatocellular Carcinoma in Patients With Hepatitis B Virus-Related Cirrhosis
Source: Front Oncol. 2022 Mar 14;12:800787. doi: 10.3389/fonc.2022.800787 (PMC8964115; doi:10.3389/fonc.2022.800787)
Supplement: Supplementary file 1 [file DataSheet_1.pdf]

frontiers

Supplementary Material

1 Supplementary material

1.1 The training, internal validation, external validation-1 or external validation-2 cohort: The included patients from First Affiliated Hospital of Guangxi University of Chinese Medicine were randomly divided into the training cohort and the internal validation cohort according to a 3:1 ratio. The included patients from Liuzhou People's Hospital and the Second Affiliated Hospital of Guangzhou University of Chinese Medicine were enrolled in the external validation-1 cohort, while the included patients from Shuguang Hospital of Shanghai University of Traditional Chinese Medicine and Affiliated Hospital of Guilin Medical University were enrolled in the external validation-2 cohort. The related clinical information of external validation-2 cohort was incomplete. So table 3 in manuscripts does not include external validation-2 cohort. This retrospective study was approved by the ethics committee, and the requirements for informed consent were waived.

1.2 Diagnosis for cirrhosis in conventional MRI and HBV cirrhosis

1.2.1 Typical features of cirrhosis in conventional MRI

(1) Morphologic changes: The cirrhotic liver develops characteristic morphologic alterations, such as surface nodularity, fibrous septa, widening of fissures, expansion of the gallbladder fossa, notching of the right lobe, atrophy of the right lobe, and relative enlargement of the lateral segments of the left lobe and caudate lobe. (2) Hepatic imaging features: In patients with advanced cirrhosis, fibrotic septa and bridges appear as low-signal-intensity reticulations on T1-weighted images and high-signal-intensity reticulations on T2-weighted images, and enhanced progressive to peak during the late venous and equilibrium phases of dynamic imaging after intravenous contrast material (most gadolinium-based contrast agents). The liver fibrotic septa surround regenerative nodules, which typically have intermediate to high signal intensity on unenhanced T1-weighted images and intermediate to low signal intensity on unenhanced T2-weighted images. (3) Extrahepatic imaging features： MRI can also show extrahepatic imaging features caused by portal hypertension, such as enlargement of the portal vein, splenomegaly, ascites and portosystemic collateral formation.

1.2.2 Diagnosis for HBV cirrhosis

The diagnostic criteria for HBV cirrhosis according to the guidelines of prevention and treatment for chronic hepatitis B (2019 version) should meet either 1 and 2 (pathological diagnosis) or 1 and 3 (clinical diagnosis) below: (1) Currently hepatitis B surface antigen (HBsAg) positive, or HBsAg negative, anti-HBC positive, and have a clear history of chronic HBV infection (HBsAg positive for more than 6 months in the past), and other causes are excluded. (2) Pathologically consistent with liver cirrhosis by liver biopsy. (3) Patients with 2 or more of the following 5 criteria, excluding non-cirrhotic portal hypertension: (i) imaging findings showing signs of cirrhosis and/or portal hypertension; (ii) Endoscopic examination showed varicose veins in esophagogastric fundus; (iii) The determination of liver hardness was consistent with cirrhosis; (iv) Serum biochemical examination showed decreased albumin level (< 35g/L) and /or prolonged PT (>3s); (v) Blood routine examination showed platelet count < 100×10^9 /L, etc.

1.3 Supplementary formula - Rad-score calculation:

Radscore =

Pre-contrast-W7.SRE*0.033+

Pre-contrast-W8.Co_Var_11*0.059+

HAP-W6.Co_Var_12*0.171+

HAP-W9.Mid*0.003+

PVP-W4.IMC2_10* -0.044+

PVP-W4.Co_Corr_5* -0.02+

PVP-W4.Co_Corr_6*0.052+

PVP-W7.Skewness* -0.024+

PVP-W7.Co_Corr_12*0.222+

PVP-W8.IMC1_5*0.085+

EP-W1.SVR*0.052+

EP-W1.Diff_entropy_10*0.005+

EP-W1.Entropy_GLCM_10*0.035+

EP-W6.Co_Corr_7*0.044+

EP-W7.Co_Corr_12*0.003+

EP-W8.Dissimilarity_5*0.021+

EP-W9.Mid*0.012+

T2WI-W7.Co_Var_2* -0.027

Abbreviation: SVR: surface-to-volume ratio; Co_Corr: correlation; Co_Var: variance; Mid: median; Skewness: Skewness; SD: spherical disproportion; IMC: information measure of correlation; Homo2:homogeneity2; MAD: mean absolute deviation

Cluster_Shade: cluster shade; Dissimilarity: Dissimilarity; Diff_entropy: difference entropy; Entropy: entropy; Mean: mean; SRE: short run emphasis;

W1-8: wavelet decompositions, W0 represents the originate plane and W1-8 represent 8 wavelet plane. The number written after the feature named as 1-13 represents the direction of the texture matrix. Mean indicates the average of the 13 directions.

pre-contrast: pre-contrast phase; HAP: hepatic arterial phase; PVP: portal venous phase; EP: equilibrium phase; T2WI: T2 weighted imaging.

2 Supplementary Figures and Tables

2.1 Supplementary Figures

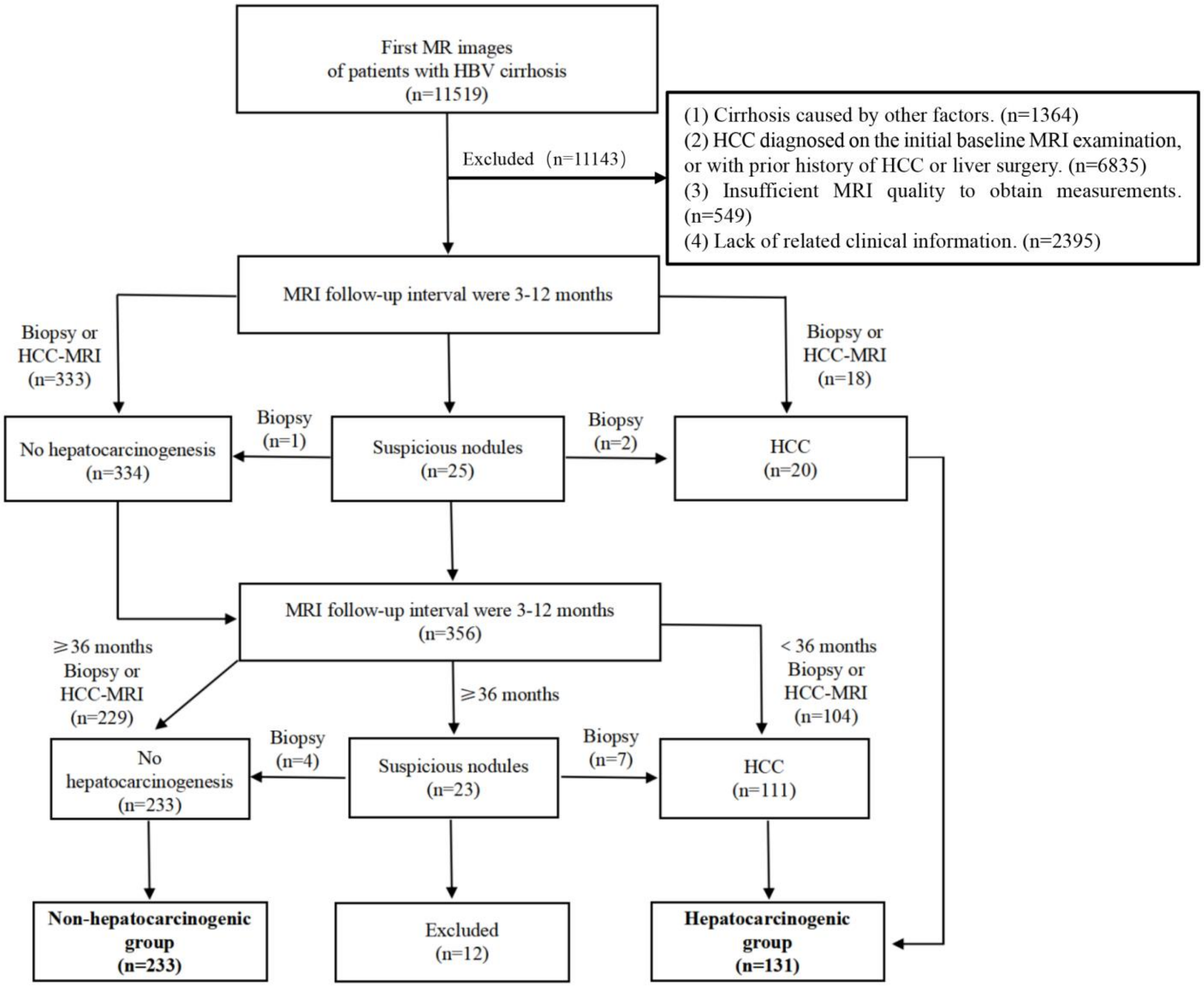

Supplementary Figure 1. HCC Surveillance and Diagnosis of patients with HBV cirrhosis are shown in the flow diagram above. Suspicious nodules on MRI including LI-RADS 3 and LI-RADS 4 according to the 2018 AASLD guidelines, lack of the typical features of imaging such as “washout” (nonperipheral) , enhancing pseudocapsule, and threshold growth.

2.2 Supplementary Tables

Supplementary Table 1. Scanner models, field strengths and acquisition settings for the five centres

	FAHGUCM[a]	SUTCM[b]	AHGM[c]	LPH[d]	SAHG[e]
Scanners	Siemens Verio 3.0T	Philips Achieva 1.5T and Siemens verio 3.0T	Siemens Avanto 1.5T and Siemens verio 3.0T	Philips Achieva 3.0T TX	Siemens Verio 3.0T and Siemens Avanto 1.5T
T_1 T_E (ms)	1.96	1.39-4.7	1.22-2.79	1.40	1.39-2.39
T_1 T_R (ms)	4.4	3.92-244	3.40-6.03	3.7	3.92-4.9
T_1 slice thickness (mm)	3.0	5-7	2-3	3.0	5-6
T_1 slice gap (mm)	0.6	1	0.4-0.6	0.6	1.2
T2 T_E (ms)	80	70-99	85-93	70	77-90
T2 T_R (ms)	2000	358-1800	3821.14-5224.72	859	2200-3000
T2 slice thickness (mm)	6	5-7	2-3	7.0	3.0
T2 slice gap (mm)	1.2	1	0.4-0.6	2.0	0.6
FOV (mm)	380×269 - 380×328	380×301-380×309	360×315-380×297	320×348-375×306	256×179-384×286

[a]FAHGUCM: The First Affiliated Hospital of Guangxi University of Chinese Medicine

[b]SUTCM: Shanghai University of Traditional Chinese Medicine

[c]AHGM: Affiliated Hospital of Guilin Medical university

[d]LPH: Liuzhou People‘s Hospital

[e]SAHG: The Second Affiliated Hospital of Guangzhou university of Chinese Medicine

Supplementary Table 2. Results of univariate Cox proportional hazards analyses for all clinical factors.

**Factors**	**p-value**
**CTP class**	**4.93×10^{-5}**
AFP	0.796
Age	0.773
**Gender**	**0.013**
FH of HCC	0.608
Smoking	0.215
HBV-DNA level	0.217
Drinking Alcohol	0.196

Abbreviations: **FH of HCC: family history of HCC; CTP class: Child-Turcotte-Pugh classification.**

Supplementary Table 3. Radiomics feature selection results based on LASSO in uni-sequence and multi-sequences MRI.

Modality	Pre-contrast	HAP	PVP	EP	T2WI	ALL
Number of features	9	4	17	5	5	18
Features list	W1.IMC2_5	W5.HGLRE_13	W1.IMC1_4	W2.SRE_4	W1.Contract_2	Pre-contrast-W7.SRE
	W2.LRLGLE_13	W7.RLN_5	W1.SRLGLE_8	W2.SRE_5	W5.Entropy_GLCM_3	Pre-contrast-W8.Co_Var_11
	W4.Max_GLCM_12	W8.Co_Var_9	W2.RP_1	W4.Max_GLCM_12	W5.Sum_var_3	HAP-W6.Co_Var_12
	W4.RLN_10	W8.Co_Var_10	W2.LRE_4	W4.Max_GLCM_13	W6.IDN_2	HAP-W9.Mid
	W6.HGLRE_12		W3.LRE_10	W8.Sum_entropy_mean	W8.Cluster_Tendency_4	PVP-W4.IMC2_10
	W6.SRLGLE_2		W4.Energy_GLCM_13			PVP-W4.Co_Corr_5
	W7.RP_12		W4.IMC2_10			PVP-W4.Co_Corr_6
	W8.AutoCorr_4		W4.IMC2_11			PVP-W7.Skewness
	W8.Diff_entropy_mean		W4.Cluster_Prominence_8			PVP-W7.Co_Corr_12
			W5.Diff_entropy_8			PVP-W8.IMC1_5
			W5.HGLRE_9			EP-W1.SVR
			W6.LRE_10			EP-W1.Diff_entropy_10
			W7.Entropy_GLCM_1			EP-W1.Entropy_GLCM_10
			W7.GLN_9			EP-W6.Co_Corr_7
			W7.LRLGLE_1			EP-W7.Co_Corr_12
			W8.Sum_entropy_1			EP-W8.Dissimilarity_5
			W8.IDNM_2			EP-W9.Mid
						T2WI-W7.Co_Var_2

Abbreviations: SVR: Surface-to-volume ratio; Co_Corr: Correlation; Co_Var: Variance; Mid: Median; Skewness: Skewness; SD: Spherical Disproportion; IMC: Information Measure of Correlation; Homo2: Homogeneity2; MAD: Mean Absolute Deviation; Cluster_Shade: Cluster Shade; RP: Run Percentage; Dissimilarity: Dissimilarity; Diff_entropy: Difference Entropy; Entropy: Entropy; Mean: Mean; GLN: Gray Level Non-Uniformity; RLN:Run Length Non-Uniformity; IDN: Inverse Difference Normalized; SRE: Short Run Emphasis; LRE: Longrun Emphasis; HGLRE: High Gray Level Run Emphasis; SRLGLE: Short Run Low Gray Level Emphasis; LRLGLE: Long Run Low Gray Level Emphasis.

pre-contrast: pre-contrast phase; HAP: hepatic arterial phase; PVP: portal venous phase; EP: equilibrium phase; T2WI: T2 weighted imaging.

W1-8: wavelet decompositions, W0 represents the originate plane and W1-8 represent 8 wavelet plane. The number written after the feature named as 1-13 represents the direction of the texture matrix. Mean indicates the average of the 13 directions.
